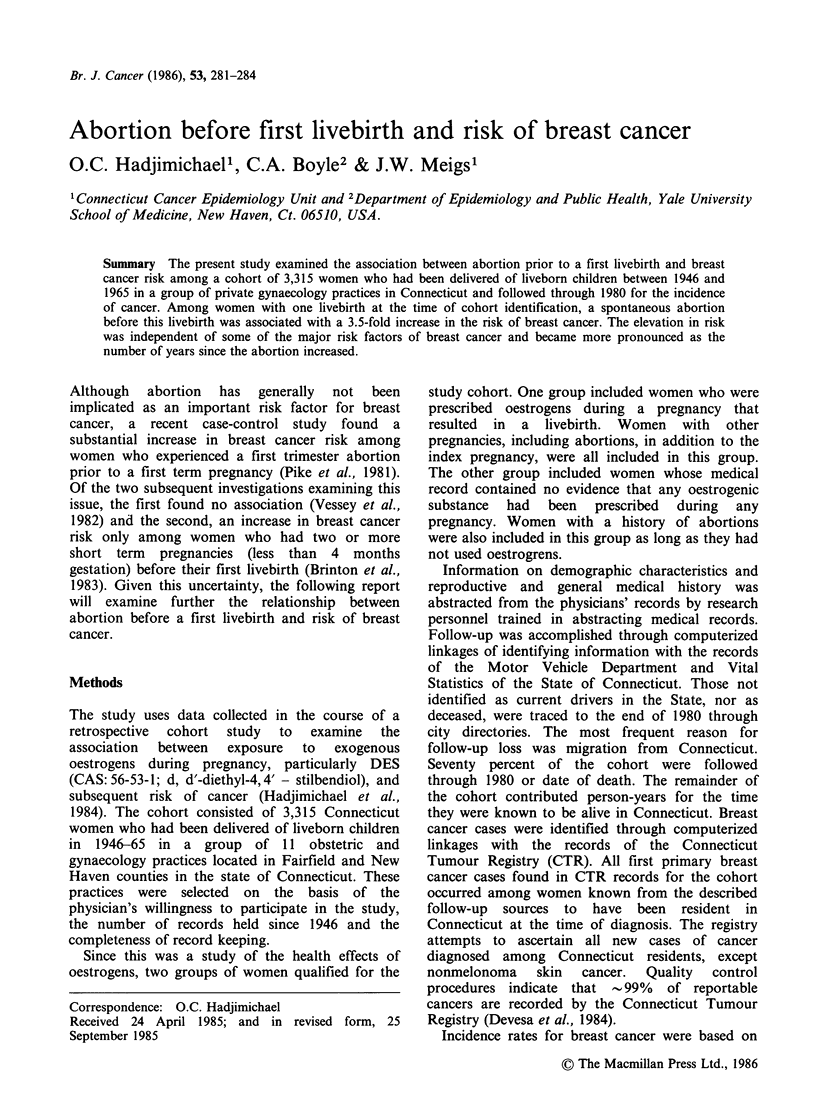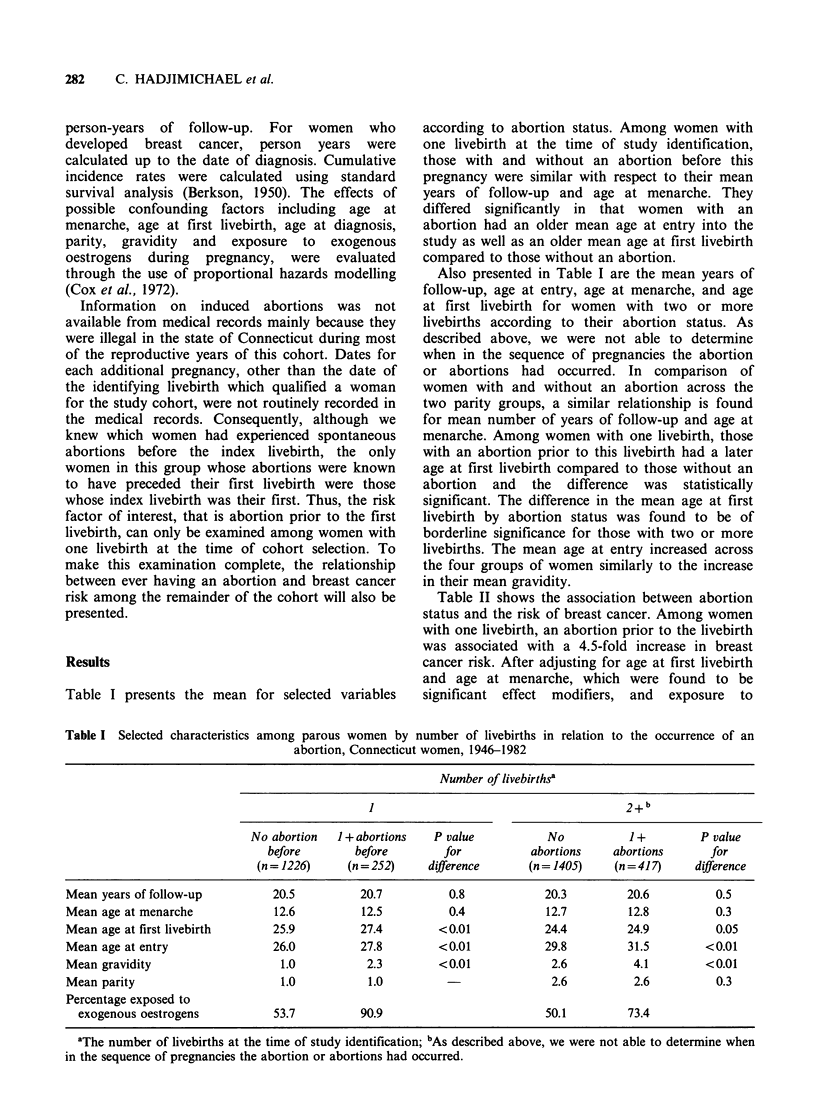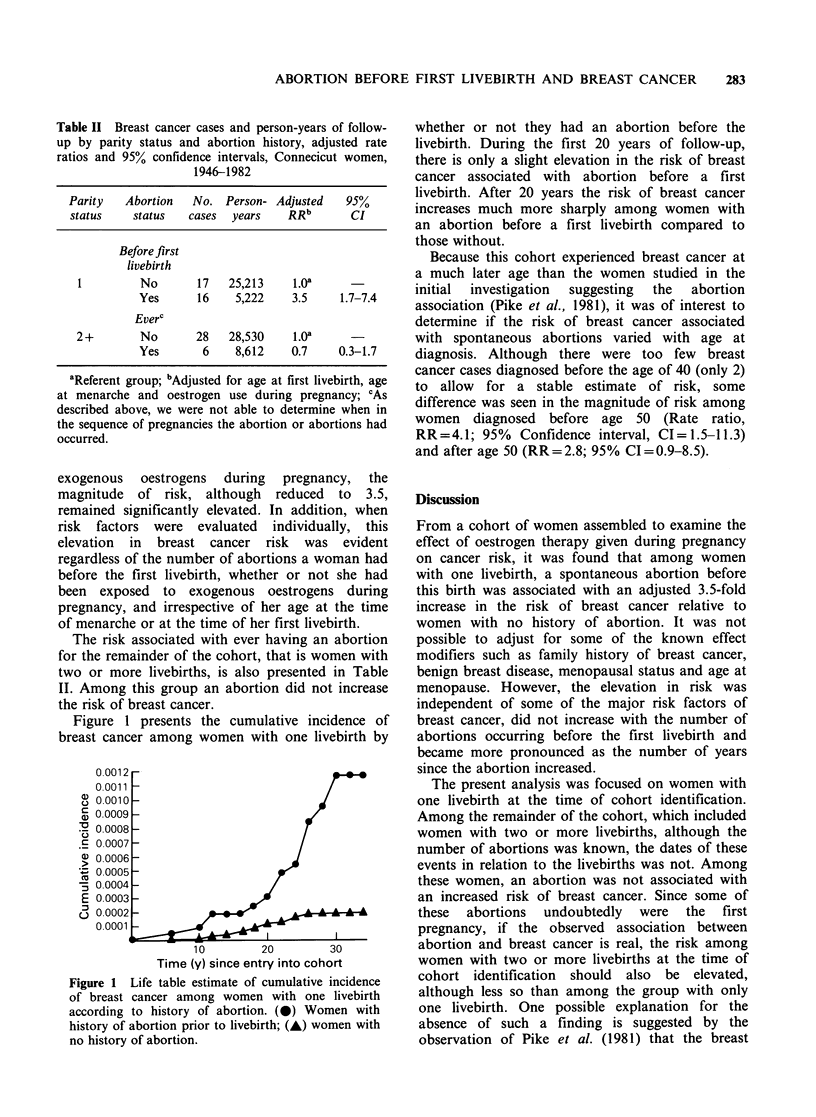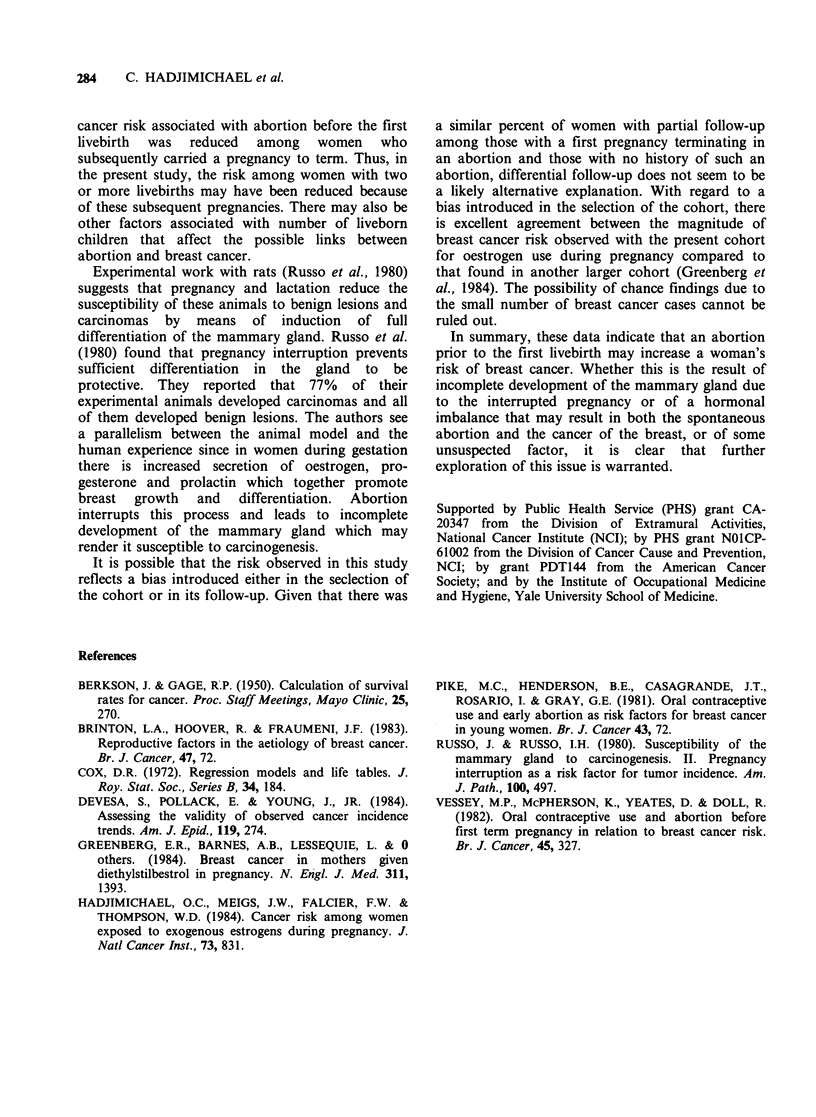# Abortion before first livebirth and risk of breast cancer.

**DOI:** 10.1038/bjc.1986.46

**Published:** 1986-02

**Authors:** O. C. Hadjimichael, C. A. Boyle, J. W. Meigs

## Abstract

The present study examined the association between abortion prior to a first livebirth and breast cancer risk among a cohort of 3,315 women who had been delivered of liveborn children between 1946 and 1965 in a group of private gynaecology practices in Connecticut and followed through 1980 for the incidence of cancer. Among women with one livebirth at the time of cohort identification, a spontaneous abortion before this livebirth was associated with a 3.5-fold increase in the risk of breast cancer. The elevation in risk was independent of some of the major risk factors of breast cancer and became more pronounced as the number of years since the abortion increased.


*Br. J. Cancer* (1986), **53**, 281–284

### Abortion before first livebirth and risk of breast cancer

O.C. Hadjimichael[1], C.A. Boyle[2] & J.W. Meigs[1]

[1]*Connecticut Cancer Epidemiology Unit and* [2]*Department of Epidemiology and Public Health, Yale University School of Medicine, New Haven, Ct. 06510, USA.*

**Summary** The present study examined the association between abortion prior to a first livebirth and breast cancer risk among a cohort of 3,315 women who had been delivered of liveborn children between 1946 and 1965 in a group of private gynaecology practices in Connecticut and followed through 1980 for the incidence of cancer. Among women with one livebirth at the time of cohort identification, a spontaneous abortion before this livebirth was associated with a 3.5-fold increase in the risk of breast cancer. The elevation in risk was independent of some of the major risk factors of breast cancer and became more pronounced as the number of years since the abortion increased.

Although abortion has generally not been implicated as an important risk factor for breast cancer, a recent case-control study found a substantial increase in breast cancer risk among women who experienced a first trimester abortion prior to a first term pregnancy (Pike *et al.*, 1981). Of the two subsequent investigations examining this issue, the first found no association (Vessey *et al.*, 1982) and the second, an increase in breast cancer risk only among women who had two or more short term pregnancies (less than 4 months gestation) before their first livebirth (Brinton *et al.*, 1983). Given this uncertainty, the following report will examine further the relationship between abortion before a first livebirth and risk of breast cancer.

### Methods

The study uses data collected in the course of a retrospective cohort study to examine the association between exposure to exogenous oestrogens during pregnancy, particularly DES (CAS: 56-53-1; d, d′-diethyl-4, 4′ – stilbendiol), and subsequent risk of cancer (Hadjimichael *et al.*, 1984). The cohort consisted of 3,315 Connecticut women who had been delivered of liveborn children in 1946–65 in a group of 11 obstetric and gynaecology practices located in Fairfield and New Haven counties in the state of Connecticut. These practices were selected on the basis of the physician's willingness to participate in the study, the number of records held since 1946 and the completeness of record keeping.

Since this was a study of the health effects of oestrogens, two groups of women qualified for the study cohort. One group included women who were prescribed oestrogens during a pregnancy that resulted in a livebirth. Women with other pregnancies, including abortions, in addition to the index pregnancy, were all included in this group. The other group included women whose medical record contained no evidence that any oestrogenic substance had been prescribed during any pregnancy. Women with a history of abortions were also included in this group as long as they had not used oestrogrens.

Information on demographic characteristics and reproductive and general medical history was abstracted from the physicians' records by research personnel trained in abstracting medical records. Follow-up was accomplished through computerized linkages of identifying information with the records of the Motor Vehicle Department and Vital Statistics of the State of Connecticut. Those not identified as current drivers in the State, nor as deceased, were traced to the end of 1980 through city directories. The most frequent reason for follow-up loss was migration from Connecticut. Seventy percent of the cohort were followed through 1980 or date of death. The remainder of the cohort contributed person-years for the time they were known to be alive in Connecticut. Breast cancer cases were identified through computerized linkages with the records of the Connecticut Tumour Registry (CTR). All first primary breast cancer cases found in CTR records for the cohort occurred among women known from the described follow-up sources to have been resident in Connecticut at the time of diagnosis. The registry attempts to ascertain all new cases of cancer diagnosed among Connecticut residents, except nonmelonoma skin cancer. Quality control procedures indicate that ~99% of reportable cancers are recorded by the Connecticut Tumour Registry (Devesa *et al.*, 1984).

Incidence rates for breast cancer were based on

Correspondence: O.C. Hadjimichael
Received 24 April 1985; and in revised form, 25 September 1985

© The Macmillan Press Ltd., 1986

person-years of follow-up. For women who developed breast cancer, person years were calculated up to the date of diagnosis. Cumulative incidence rates were calculated using standard survival analysis (Berkson, 1950). The effects of possible confounding factors including age at menarche, age at first livebirth, age at diagnosis, parity, gravidity and exposure to exogenous oestrogens during pregnancy, were evaluated through the use of proportional hazards modelling (Cox *et al.*, 1972).

Information on induced abortions was not available from medical records mainly because they were illegal in the state of Connecticut during most of the reproductive years of this cohort. Dates for each additional pregnancy, other than the date of the identifying livebirth which qualified a woman for the study cohort, were not routinely recorded in the medical records. Consequently, although we knew which women had experienced spontaneous abortions before the index livebirth, the only women in this group whose abortions were known to have preceded their first livebirth were those whose index livebirth was their first. Thus, the risk factor of interest, that is abortion prior to the first livebirth, can only be examined among women with one livebirth at the time of cohort selection. To make this examination complete, the relationship between ever having an abortion and breast cancer risk among the remainder of the cohort will also be presented.

### Results

Table I presents the mean for selected variables according to abortion status. Among women with one livebirth at the time of study identification, those with and without an abortion before this pregnancy were similar with respect to their mean years of follow-up and age at menarche. They differed significantly in that women with an abortion had an older mean age at entry into the study as well as an older mean age at first livebirth compared to those without an abortion.

Also presented in Table I are the mean years of follow-up, age at entry, age at menarche, and age at first livebirth for women with two or more livebirths according to their abortion status. As described above, we were not able to determine when in the sequence of pregnancies the abortion or abortions had occurred. In comparison of women with and without an abortion across the two parity groups, a similar relationship is found for mean number of years of follow-up and age at menarche. Among women with one livebirth, those with an abortion prior to this livebirth had a later age at first livebirth compared to those without an abortion and the difference was statistically significant. The difference in the mean age at first livebirth by abortion status was found to be of borderline significance for those with two or more livebirths. The mean age at entry increased across the four groups of women similarly to the increase in their mean gravidity.

Table II shows the association between abortion status and the risk of breast cancer. Among women with one livebirth, an abortion prior to the livebirth was associated with a 4.5-fold increase in breast cancer risk. After adjusting for age at first livebirth and age at menarche, which were found to be significant effect modifiers, and exposure to

**Table I** Selected characteristics among parous women by number of livebirths in relation to the occurrence of an abortion, Connecticut women, 1946–1982

| | *Number of livebirths*[a] | | | | | |
|---|---|---|---|---|---|---|
| | *1* | | | *2+*[b] | | |
| | *No abortion before (n=1226)* | *1+abortions before (n=252)* | *P value for difference* | *No abortions (n=1405)* | *1+ abortions (n=417)* | *P value for difference* |
| Mean years of follow-up | 20.5 | 20.7 | 0.8 | 20.3 | 20.6 | 0.5 |
| Mean age at menarche | 12.6 | 12.5 | 0.4 | 12.7 | 12.8 | 0.3 |
| Mean age at first livebirth | 25.9 | 27.4 | <0.01 | 24.4 | 24.9 | 0.05 |
| Mean age at entry | 26.0 | 27.8 | <0.01 | 29.8 | 31.5 | <0.01 |
| Mean gravidity | 1.0 | 2.3 | <0.01 | 2.6 | 4.1 | <0.01 |
| Mean parity | 1.0 | 1.0 | — | 2.6 | 2.6 | 0.3 |
| Percentage exposed to exogenous oestrogens | 53.7 | 90.9 | | 50.1 | 73.4 | |

[a]The number of livebirths at the time of study identification; [b]As described above, we were not able to determine when in the sequence of pregnancies the abortion or abortions had occurred.

**Table II** Breast cancer cases and person-years of follow-up by parity status and abortion history, adjusted rate ratios and 95% confidence intervals, Connecicut women, 1946–1982

| *Parity status* | *Abortion status* | *No. cases* | *Person-years* | *Adjusted RR*[b] | *95% CI* |
|---|---|---|---|---|---|
| | *Before first livebirth* | | | | |
| 1 | No | 17 | 25,213 | 1.0[a] | — |
| | Yes | 16 | 5,222 | 3.5 | 1.7–7.4 |
| | *Ever*[c] | | | | |
| 2+ | No | 28 | 28,530 | 1.0[a] | — |
| | Yes | 6 | 8,612 | 0.7 | 0.3–1.7 |

[a]Referent group; [b]Adjusted for age at first livebirth, age at menarche and oestrogen use during pregnancy; [c]As described above, we were not able to determine when in the sequence of pregnancies the abortion or abortions had occurred.

exogenous oestrogens during pregnancy, the magnitude of risk, although reduced to 3.5, remained significantly elevated. In addition, when risk factors were evaluated individually, this elevation in breast cancer risk was evident regardless of the number of abortions a woman had before the first livebirth, whether or not she had been exposed to exogenous oestrogens during pregnancy, and irrespective of her age at the time of menarche or at the time of her first livebirth.

The risk associated with ever having an abortion for the remainder of the cohort, that is women with two or more livebirths, is also presented in Table II. Among this group an abortion did not increase the risk of breast cancer.

Figure 1 presents the cumulative incidence of breast cancer among women with one livebirth by

**Figure 1** Life table estimate of cumulative incidence of breast cancer among women with one livebirth according to history of abortion. (●) Women with history of abortion prior to livebirth; (▲) women with no history of abortion.

whether or not they had an abortion before the livebirth. During the first 20 years of follow-up, there is only a slight elevation in the risk of breast cancer associated with abortion before a first livebirth. After 20 years the risk of breast cancer increases much more sharply among women with an abortion before a first livebirth compared to those without.

Because this cohort experienced breast cancer at a much later age than the women studied in the initial investigation suggesting the abortion association (Pike *et al.*, 1981), it was of interest to determine if the risk of breast cancer associated with spontaneous abortions varied with age at diagnosis. Although there were too few breast cancer cases diagnosed before the age of 40 (only 2) to allow for a stable estimate of risk, some difference was seen in the magnitude of risk among women diagnosed before age 50 (Rate ratio, RR=4.1; 95% Confidence interval, CI=1.5–11.3) and after age 50 (RR=2.8; 95% CI=0.9–8.5).

### Discussion

From a cohort of women assembled to examine the effect of oestrogen therapy given during pregnancy on cancer risk, it was found that among women with one livebirth, a spontaneous abortion before this birth was associated with an adjusted 3.5-fold increase in the risk of breast cancer relative to women with no history of abortion. It was not possible to adjust for some of the known effect modifiers such as family history of breast cancer, benign breast disease, menopausal status and age at menopause. However, the elevation in risk was independent of some of the major risk factors of breast cancer, did not increase with the number of abortions occurring before the first livebirth and became more pronounced as the number of years since the abortion increased.

The present analysis was focused on women with one livebirth at the time of cohort identification. Among the remainder of the cohort, which included women with two or more livebirths, although the number of abortions was known, the dates of these events in relation to the livebirths was not. Among these women, an abortion was not associated with an increased risk of breast cancer. Since some of these abortions undoubtedly were the first pregnancy, if the observed association between abortion and breast cancer is real, the risk among women with two or more livebirths at the time of cohort identification should also be elevated, although less so than among the group with only one livebirth. One possible explanation for the absence of such a finding is suggested by the observation of Pike *et al.* (1981) that the breast cancer risk associated with abortion before the first livebirth was reduced among women who subsequently carried a pregnancy to term. Thus, in the present study, the risk among women with two or more livebirths may have been reduced because of these subsequent pregnancies. There may also be other factors associated with number of liveborn children that affect the possible links between abortion and breast cancer.

Experimental work with rats (Russo *et al.*, 1980) suggests that pregnancy and lactation reduce the susceptibility of these animals to benign lesions and carcinomas by means of induction of full differentiation of the mammary gland. Russo *et al.* (1980) found that pregnancy interruption prevents sufficient differentiation in the gland to be protective. They reported that 77% of their experimental animals developed carcinomas and all of them developed benign lesions. The authors see a parallelism between the animal model and the human experience since in women during gestation there is increased secretion of oestrogen, progesterone and prolactin which together promote breast growth and differentiation. Abortion interrupts this process and leads to incomplete development of the mammary gland which may render it susceptible to carcinogenesis.

It is possible that the risk observed in this study reflects a bias introduced either in the seclection of the cohort or in its follow-up. Given that there was a similar percent of women with partial follow-up among those with a first pregnancy terminating in an abortion and those with no history of such an abortion, differential follow-up does not seem to be a likely alternative explanation. With regard to a bias introduced in the selection of the cohort, there is excellent agreement between the magnitude of breast cancer risk observed with the present cohort for oestrogen use during pregnancy compared to that found in another larger cohort (Greenberg *et al.*, 1984). The possibility of chance findings due to the small number of breast cancer cases cannot be ruled out.

In summary, these data indicate that an abortion prior to the first livebirth may increase a woman's risk of breast cancer. Whether this is the result of incomplete development of the mammary gland due to the interrupted pregnancy or of a hormonal imbalance that may result in both the spontaneous abortion and the cancer of the breast, or of some unsuspected factor, it is clear that further exploration of this issue is warranted.

Supported by Public Health Service (PHS) grant CA-20347 from the Division of Extramural Activities, National Cancer Institute (NCI); by PHS grant N01CP-61002 from the Division of Cancer Cause and Prevention, NCI; by grant PDT144 from the American Cancer Society; and by the Institute of Occupational Medicine and Hygiene, Yale University School of Medicine.

### References

BERKSON, J. & GAGE, R.P. (1950). Calculation of survival rates for cancer. *Proc. Staff Meetings, Mayo Clinic*, **25**, 270.

BRINTON, L.A., HOOVER, R. & FRAUMENI, J.F. (1983). Reproductive factors in the aetiology of breast cancer. *Br. J. Cancer*, **47**, 72.

COX, D.R. (1972). Regression models and life tables. *J. Roy. Stat. Soc., Series B*, **34**, 184.

DEVESA, S., POLLACK, E. & YOUNG, J., JR. (1984). Assessing the validity of observed cancer incidence trends. *Am. J. Epid.*, **119**, 274.

GREENBERG, E.R., BARNES, A.B., LESSEQUIE, L. & 0 others. (1984). Breast cancer in mothers given diethylstilbestrol in pregnancy. *N. Engl. J. Med.* **311**, 1393.

HADJIMICHAEL, O.C., MEIGS, J.W., FALCIER, F.W. & THOMPSON, W.D. (1984). Cancer risk among women exposed to exogenous estrogens during pregnancy. *J. Natl Cancer Inst.*, **73**, 831.

PIKE, M.C., HENDERSON, B.E., CASAGRANDE, J.T., ROSARIO, I. & GRAY, G.E. (1981). Oral contraceptive use and early abortion as risk factors for breast cancer in young women. *Br. J. Cancer* **43**, 72.

RUSSO, J. & RUSSO, I.H. (1980). Susceptibility of the mammary gland to carcinogenesis. II. Pregnancy interruption as a risk factor for tumor incidence. *Am. J. Path.*, **100**, 497.

VESSEY, M.P., McPHERSON, K., YEATES, D. & DOLL, R. (1982). Oral contraceptive use and abortion before first term pregnancy in relation to breast cancer risk. *Br. J. Cancer*, **45**, 327.